# A Mathematical Model of Bimodal Epigenetic Control of miR-193a in Ovarian Cancer Stem Cells

**DOI:** 10.1371/journal.pone.0116050

**Published:** 2014-12-29

**Authors:** Frank H. C. Cheng, Baltazar D. Aguda, Je-Chiang Tsai, Marek Kochańczyk, Jora M. J. Lin, Gary C. W. Chen, Hung-Cheng Lai, Kenneth P. Nephew, Tzy-Wei Hwang, Michael W. Y. Chan

**Affiliations:** 1 Department of Life Science, National Chung Cheng University, Min-Hsiung, Chia-Yi, Taiwan, Republic of China; 2 Institute of Molecular Biology, National Chung Cheng University, Min-Hsiung, Chia-Yi, Taiwan, Republic of China; 3 Department of Mathematics, National Chung Cheng University, Min-Hsiung, Chia-Yi, Taiwan, Republic of China; 4 DiseasePathways LLC, Bethesda, Maryland, United States of America; 5 Institute of Fundamental Technological Research, Polish Academy of Sciences, Warsaw, Poland; 6 Department of Obstetrics and Gynecology, School of Medicine, College of Medicine, Taipei Medical University, Taipei, Taiwan, Republic of China; 7 Department of Obstetrics and Gynecology, Shuang Ho Hospital, Taipei Medical University, Taipei, Taiwan, Republic of China; 8 Department of Clinical Pharmacology, Xiangya Hospital, Central South University, Changsha, People's Republic of China; 9 Institute of Clinical Pharmacology, Central South University, Changsha, People's Republic of China; 10 Hunan Key Laboratory of Pharmacogenetics, Changsha, People's Republic of China; 11 Medical Sciences, Department of Cellular and Integrative Physiology, Indiana University School of Medicine, Bloomington, Indiana, United States of America; The University of Hong Kong, Hong Kong

## Abstract

Accumulating data indicate that cancer stem cells contribute to tumor chemoresistance and their persistence alters clinical outcome. Our previous study has shown that ovarian cancer may be initiated by ovarian cancer initiating cells (OCIC) characterized by surface antigen CD44 and c-KIT (CD117). It has been experimentally demonstrated that a microRNA, namely miR-193a, targets c-KIT mRNA for degradation and could play a crucial role in ovarian cancer development. How miR-193a is regulated is poorly understood and the emerging picture is complex. To unravel this complexity, we propose a mathematical model to explore how estrogen-mediated up-regulation of another target of miR-193a, namely E2F6, can attenuate the function of miR-193a in two ways, one through a competition of E2F6 and c-KIT transcripts for miR-193a, and second by binding of E2F6 protein, in association with a polycomb complex, to the promoter of miR-193a to down-regulate its transcription. Our model predicts that this bimodal control increases the expression of c-KIT and that the second mode of epigenetic regulation is required to generate a switching behavior in c-KIT and E2F6 expressions. Additional analysis of the TCGA ovarian cancer dataset demonstrates that ovarian cancer patients with low expression of EZH2, a polycomb-group family protein, show positive correlation between E2F6 and c-KIT. We conjecture that a simultaneous EZH2 inhibition and anti-estrogen therapy can constitute an effective combined therapeutic strategy against ovarian cancer.

## Introduction

Ovarian cancer is the most lethal gynecological malignancy and the 5th leading cause of cancer death among women [1]. As ovarian cancer has few symptoms early in its course, the majority of patients are diagnosed with late stages (III and IV) of the disease. The 5-year survival rate is generally less than 20% for patients with advanced-stage disease despite therapeutic advances, whereas survival rate for patients with stage I or II disease is greater than 80% for the same period [2]. Although current chemotherapeutics demonstrate a more than 90% complete response rate in early-stage tumors, only a 20–30% partial response rate is observed in advanced stage tumors as well as in chemo-resistant relapsed tumors [3]. A better understanding of the molecular changes of ovarian carcinogenesis may lead to better therapeutic strategies for this deadly disease.

An emerging hypothesis states that cancer arises from a small population of self-renewing cancer initiating cells (CIC) [4]. These CICs are thought to possess tumorigenic potential and enhanced drug resistance within a tumor, and are able to repopulate tumor colonies *in vivo*. We have previously isolated ovarian CICs (OCICs) from ovarian cancer patients [5]. These OCICs exhibit increased drug resistance towards cisplatin and taxol, and are characterized by the expression of several cell surface markers, including c-KIT (CD117, or stem cell factor receptor). However, the mechanism of how OCICs arise and how these cell surface markers are transcriptionally controlled are not fully understood.

Estrogen is an important regulator of growth and differentiation in normal ovaries [6] and is involved in the development of ovarian cancer [7]. In this regard, studies have explored the risk of ovarian cancer among women using hormone replacement therapy (HRT) in the treatment of menopausal symptoms [8], [9], [10]. For example, a study involving a million women suggested that women treated with HRT were associated with an increased risk of ovarian cancer [11]. However, the role of estrogen in ovarian carcinogenesis is not fully understood.

Epigenetic modifications in the genome play a crucial role in transcriptional control and aberrant epigenetic modification is now considered a hallmark of cancer [12]. Epigenetic modifications are responsible for controlling gene expressions that allow for specific phenotypes [13], [14]. Among them, DNA methylation is one of the most characterized epigenetic modification which takes place at the 5′ position of cytosine in CpG dinucleotides resulting in the formation of 5-methylcytosine. DNA methylation which occurs at the clusters of CpG sites (called CpG islands) in the promoter region of a tumor suppressor gene results in transcriptional repression and tumorigenesis in some cases [15]. Also considered as crucial regulators of gene expression, miRs are referred to as tumor suppressor-miRs if they regulate the expression of an oncogene [14]. Studies found that tumor suppressor-miRs are frequently down-regulated by genetic or epigenetic mechanisms leading to the up-regulation of oncogenes and acceleration of tumorigenesis [16]. For example, miR-34b/c (which resides at the promoter CpG island of a gene) was found to be epigenetically silenced by DNA methylation in metastatic cancer cells [17]. Re-expression of miR-34b/c suppressed cancer invasion *in vitro* and *in vivo*.

We are interested in miR-193a which is located at chromosome 17q11.2 and embedded in a CpG island. Studies have found that miR-193a was epigenetically silenced by promoter hypermethylation in acute myeloid leukemia (AML) and lung cancer [18], [19]. Importantly, miR-193a targets the stem cell marker, c-KIT, for repression in AML [18], [20]. Another experimentally confirmed target of miR-193a is E2F6 [21]. Up-regulation of E2F6 has been observed in breast cancer cells upon treatment with estrogen [22]. Within the E2F family of transcription factors which are involved in cell cycle control through transcriptional activation or repression [23], E2F6 was found to be a transcriptional repressor capable of associating with the histone-lysine N-methyltransferase EZH2, which is an important constituent of the polycomb complex [24], [25], and with DNA methyl-transferase DNMT3b, allowing for its recruitment to the repressive complex [26].

As the role of miR-193a in ovarian cancer is currently unknown, we hypothesize that E2F6 protein may suppress the expression of miR-193a, both directly as a DNA-binding transcriptional repressor and indirectly through promotion of the polycomb complex assembly. Indeed, the existence of the binding sites for E2F6 protein in the promoter of miR-193a as demonstrated by ENCODE ChIP-Seq data [27] seems to suggest that long-term suppression of miR-193a by E2F6 may lead to epigenetic silencing of miR-193a. These events may lead to the up-regulation of c-KIT and trigger the onset of the OCIC phenotype, thus promoting ovarian carcinogenesis. In this study, we use a mathematical model to explore the intricacies of this epigenetic control of miR-193a which could prove to be significant as it predicts a switching behavior in c-KIT that could be critical for ovarian carcinogenesis.

## Materials and Methods

### Cell culture

Ovarian cancer cell line A2780 was propagated in RPMI1640 (Invitrogen, Carlsbad, CA) supplemented with 10% FBS, and 50 units/ml of penicillin/streptomycin. The cell was incubated at 37°Cwith 5% CO_2_.

### Cloning of miR-193a expressing vector

The sequences which transcribe miR-193a pre-miRNA was amplified by PCR using specific primers F: 5′-CGCGGATCCAGTTTCTCGGCGCATAACTC-3′ and R: 5′-CCCAAGCTTCGCTATTTCTCCAGCGAAGTG-3′, using genomic DNA of IOSE cells which express miR-193a. The PCR products were ligated into yT&A Cloning Vector (Yeastern Biotech, Taiwan) for sequencing confirmation. miR-193a-yT&A was digested with BamH I and Hind III, and inserted into multiple cloning site of p*Silencer* 4.1-CMV puro siRNA expression vector, which was predigested with BamHI and HindIII.

### Extraction of RNA and Quantitative RT-PCR

RNA extraction was performed using TRIzol reagent (Invitrogen Carlsbad, CA) according to the manufacturer’s protocol. To remove potential contaminating DNA from the complementary DNA, 1 µg of total RNA was treated with DNase I (Amplification Grade, Invitrogen) prior to reverse transcription. RNA was then reverse-transcribed using random primers (for c-KIT or E2F6 expression) or TaqMan reverse transcription kit (Applied Biosystems, Foster City, CA, USA). Quantitative real-time RT-PCR was then performed using ABI StepOne real-time PCR system (Applied Biosystems). Primers are available upon request. Relative expression of miR-193a, c-KIT and E2F6 was calculated using the comparative Ct method.

## Results

### miR-193a targets c-KIT and E2F6

**Figure 1:**
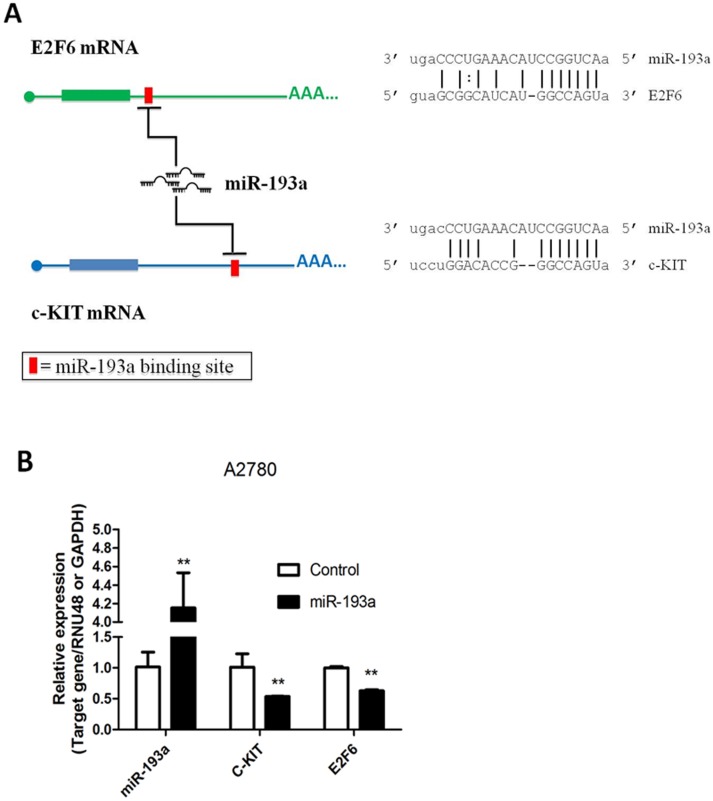
miR-193a repress E2F6 and c-KIT. (A) Schematic diagram of c-KIT and E2F6 mRNA showing predicted miR-193a binding site at their 3′UTR. The detailed binding sequences (MREs) of these sites are also shown along with miRNA sequence alignments. (B) qPCR analysis of miR-193a, c-KIT and E2F6 in A2780 ovarian cancer cells. Overexpression of miR-193a in A2780 cells leads to down-regulation of c-KIT and E2F6 mRNAs.

To investigate the relationship between miR-193a and c-KIT/E2F6 in ovarian cancer (Fig. 1A), we over-expressed miR-193a in A2780 ovarian cancer cells which have low levels of miR-193a expression. Expression of miR-193a resulted in a significant down-regulation of c-KIT mRNA and E2F6 mRNA, thus suggesting that c-KIT mRNA and E2F6 mRNA are targets of miR-193a in ovarian cancer cells (Fig. 1B).

### Model equations

**Figure 2:**
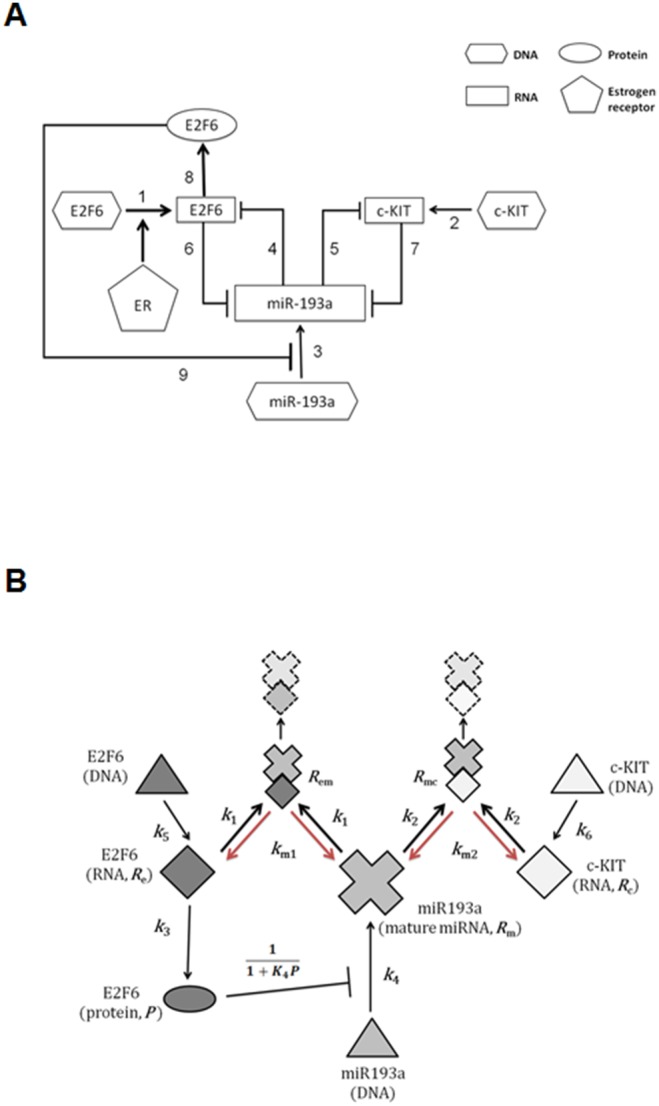
The proposed miR-193a regulatory network. (A) Activation of estrogen receptor (ER) signaling may increase the increase the transcription of E2F6 which may subsequently increase the expression of c-KIT through miR-193a mediated ceRNA mechanism. On the other hand, in addition to transcriptional repressor, E2F6 may lead to epigenetic silencing of the miR-193a promoter resulting in the up-regulation of c-KIT. The description for each number in the pathway is listed in S1 Table. (B) The biochemical interaction for this proposed miR-193a regulatory network. Model variables names for molecule species are also given.

Interactions between the components of the miR-193a–E2F6–c-KIT regulatory module are shown in Fig. 2A. Tight coupling of network components and potential non-linear kinetics can give rise to complex system dynamics, which can be understood with the help of a mathematical model. In our kinetic model (Fig. 2B) there are six components, which represent: miR-193a (*R*_m_), E2F6 mRNA (*R*_e_), c-KIT mRNA (*R*_c_), the complex of miR-193a with E2F6 mRNA (*R*_em_), complex of miR-193a with c-KIT mRNA (*R*_mc_), and E2F6 protein (*P*). The chemical mass-action equations for the respective concentration of these molecules or complexes, *R*_m_, *R*_e_, *R*_c_, *R*_em_, *R*_mc_, and *P*, are stated as follows:
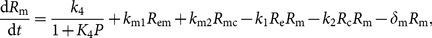
(1)

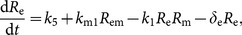
(2)

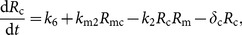
(3)

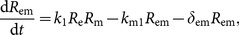
(4)

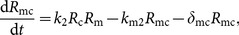
(5)

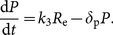
(6)


Here, the association rate of miR-193a–E2F6 mRNA is *k*_1_ and association rate of miR-193a–c-KIT mRNA is *k*_2_; the dissociation rate of complexes miR-193a–E2F6 mRNA is *k*_m1_ and miR-193a–c-KIT mRNA is *k*_m2_; the translation rate of E2F6 protein is *k*_3_; the transcription rate of miR-193a is *k*_4_, of E2F6 mRNA is *k*_5_ and of c-KIT mRNA is *k*_6_; the degradation rate of miR-193a is *δ*_m_, of E2F6 mRNA is *δ*_e_, of c-KIT mRNA is *δ*_c_, of miR-193a–E2F6 mRNA is *δ*_em_, and of miR-193a–c-KIT mRNA is *δ*_mc_ and the degradation rate of E2F6 protein is *δ*_p_; and the strength of the miR-193a transcription inhibition by E2F6 protein is denoted as *K*_4_.

The first term on the right-hand side of Eq. (1) corresponds to arrow 9 in Fig. 2A expressing miR-193a transcription inhibition by, primarily, E2F6 protein, but also by polycomb complex proteins activity. The value of the parameter *K*_4_ is thus intended to account for both the direct transcription repression by E2F6 and for the long-term gene silencing performed by and dependent of the levels of EZH2 and DNMT3b. (The concentration of E2F6 is a variable of the dynamical model while levels of essential polycomb proteins, which are not explicitly present in model equations, are not subjects to regulation by the module and their levels are assumed constant at least at the module equilibration time scale.) The second (respectively, third) term on the right-hand side of Eq. (1) is the rate of miR-193a release from the complex with E2F6 mRNA (respectively, the complex with c-KIT mRNA). Interactions corresponding to arrows 6 and 7 in Fig. 2A, expressing hybrid mRNA complex formation, are given, respectively, by the fourth and the fifth term on the right-hand side of Eq. (1). The last term in Eq. (1) is a first-order miR-193a degradation term.

The rate of constitutive E2F6 gene transcription (arrow 1 in Fig. 2A) is *k*_5_ (Eq. (2)). This rate depends on the level of hormonal stimulation and, as our model describes simplified regulation in ovarian cells, we will directly associate *k*_5_ with the level of estrogen. The second term in Eq. (2) represents the release of E2F6 mRNA from the complex with miR-193a, whereas the third term is the rate of the opposite binding reaction. The last term in Eq. (2) is a degradation of E2F6 mRNA. The terms on the right-hand side of Eq. (3) are analogous. The first term in Eq. (4) is the rate for the binding of E2F6 mRNA and miR-193a. The second term in Eq. (4) stands for the opposite reaction, and the third term represents the degradation of *R*_em_. The terms on the right-hand side of Eq. (5) are analogous. The last equation accounts for E2F6 translation (arrow 8 in Fig. 2A) and degradation.

### Steady States

Steady states give the long-term behavior of the system. The steady states of system (1)–(6) can be obtained by equating the right-hand sides of the equations to zero. Let *S* = (*R*_m_, *R*_e_, *R*_c_, *R*_em_, *R*_mc_, *P*), be any steady state of system (1)–(6). The steady state *S* satisfies the following equations
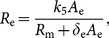
(7)

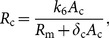
(8)

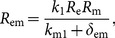
(9)

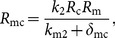
(10)

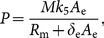
(11)

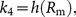
(12)where



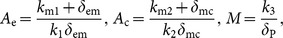



and
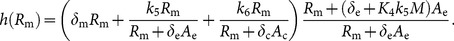
(13)


We note that once *R*_m_ is determined by Eq. (12), then the other components of the steady state *S* can be determined by Eqs. (7)–(11). Hence the condition for the existence of the steady state *S* is that *k*_4_ lies in *h*[0, ∞), the range of the function *h*. We are interested in threshold or switching behavior of system (1)–(6), particularly in switching of the expression of c-KIT mRNA. Thus, the conditions on the parameters for the existence of multiple steady states are relevant. Here we give a criterion for the existence of three steady states of the model as follows:
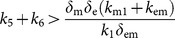
(14)

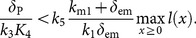
(15)where 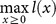 is the maximal value of 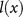 on the interval [0, ∞), and 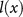 is given by



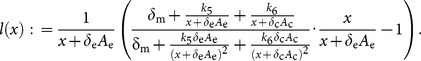



The proof for the criterion (14)–(15) is given in the S1 Text.

### The Steady State Bifurcation Diagram in the (*k*_5_, *R*_c_) plane

In view of Eqs. (7)–(8), the steady states of *R*_e_ and *R*_c_ increase or decrease in the same direction. This model prediction is consistent with the ceRNA hypothesis [28], [29] that the expression of E2F6 positively correlates with that of c-KIT.

**Figure 3:**
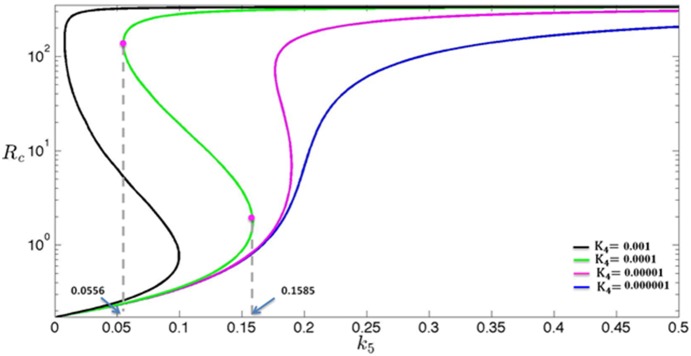
Steady-state bifurcation diagrams. The dependence of the steady states of variable *R*_c_ (RNA level of c-KIT) on parameter *k*_5_ (transcription rate of E2F6) for difference values of the parameter *K*_4_ (inhibition efficiency of E2F6). The value of parameters are listed in S2 Text.

As mentioned before, *k*_5_ is an experimentally controllable parameter associated with the addition of hormones (e.g., estrogen), and *K*_4_ is a measure of the inhibition efficiency of, primarily, E2F6 protein against miR-193a. As we will see in the discussion below, the interplay between *k*_5_ and *K*_4_ can generate an over-expression of c-KIT mRNA. Hence, it would be interesting to see the dependence of the steady state on the parameter *k*_5_ for different values of *K*_4_ – this is shown in Fig. 3. This diagram is referred to as a “steady-state bifurcation diagrams”, and *k*_5_ is referred to as a bifurcation parameter. To generate experimental curves similar to those shown in Fig. 3, one can design an experiment in which cells are cultured at different concentrations of estrogen, and then measure the long-term mRNA levels.

With parameters satisfying Eq. (14)–(15), the model predicts that there is a range of *k*_5_ for which the system admits three coexisting steady states. For instance, for *K*_4_ = 0.0001 (i.e., a lower inhibition efficiency; Fig. 3, green line) and *k*_5_ is between 0.0556 and 0.1585, the model gives three steady states (out of which the high *R*_c_ and the low *R*_c_ states are stable; see the curve associated with *K*_4_ = 0.0001 in Fig. 3). The importance of the right knee in a steady-state bifurcation diagram lies in the following fact: as the control parameter *k*_5_ is increased from the low value up through the critical value *k*_5_^R^ associated with the right knee, the corresponding steady state level of *R*_c_ will increase and, due to the instability (respectively, stability) of the steady states on the middle branch (respectively, the upper branch) in the steady-state bifurcation diagram, the system will jump to the steady state on the upper branch at the critical value *k*_5_^R^, and then stay on the upper branch (see Fig. 3). There is a sharp difference in c-KIT mRNA levels between the steady states of *R*_c_ on the lower and upper branches in the steady-state bifurcation diagram, which would suggest cancer phenotype-promoting over-expression of c-KIT mRNA for *k*_5_>*k*_5_^R^. On the other hand, if one is initially at the steady state on the upper branch which is associated with the over expression of c-KIT mRNA, one can decrease the *k*_5_ value to reduce the steady state level of *R*_c_. Moreover, if one continues to decrease the *k*_5_ value below the critical value *k*_5_^L^associated with the left knee, then the corresponding steady state level of *R*_c_ will decrease and, due to the instability (respectively, stability) of the steady states on the middle branch (respectively, the lower branch) in the steady-state bifurcation diagram, the system will descend to the steady state on the lower branch at the critical value *k*_5_^L^ (see Fig. 3). This would recover the normal c-KIT mRNA level.

To summarize, the model predicts that there exists a threshold value *k*_5_^R^ (respectively, *k*_5_^L^) in the transcriptional rate *k*_5_ for cells to turn on (respectively, turn off) the expression of OCIC marker c-KIT. Hence, it is important to adjust the transcriptional rate *k*_5_ to affect the expression of OCIC marker c-KIT.

### Significance of the Parameter *K*_4_

Although decreasing the value of *k*_5_ (associated with low estrogen level) can reduce the expression of the OCIC marker c-KIT, some clinical evidence suggests that anti-estrogen therapy is only partially effective in the treatment of ovarian cancer [30], [31]. The model suggests that this might be due to the inhibition of miR-193a expression by E2F6 protein as reflected in the value of the parameter *K*_4_ of the first term on the right-hand side of Eq. (1). This term is motivated by the fact that the expression of miR-193a depends on the transcriptional suppressive activity of E2F6 protein (associated with the level of EZH2 and DNMTs). In particular, sufficiently strong inhibition (sufficiently large *K*_4_) would lead to a more long-term silencing of the expression of miR-193a by DNA methylation (see Eq. (1)), and thus would entail the over-expression of c-KIT mRNA; whereas for the extreme case *K*_4_ = 0, the system (1)–(6) can only support at most one steady state (see S1 Text), which implies the non-existence of the right and left knees in the steady state diagram and loss of the bistable switch.

Let *k*_5_^b^ denote the basal transcription rate constant of E2F6 mRNA. In the following, depending on the interplay between the *k*_5_^b^ and *K*_4_, we will describe two scenarios in which the second one would correspond to the clinical observation that anti-estrogen therapy are only partially effective in the treatment of ovarian cancer [30], [31].

**Figure 4:**
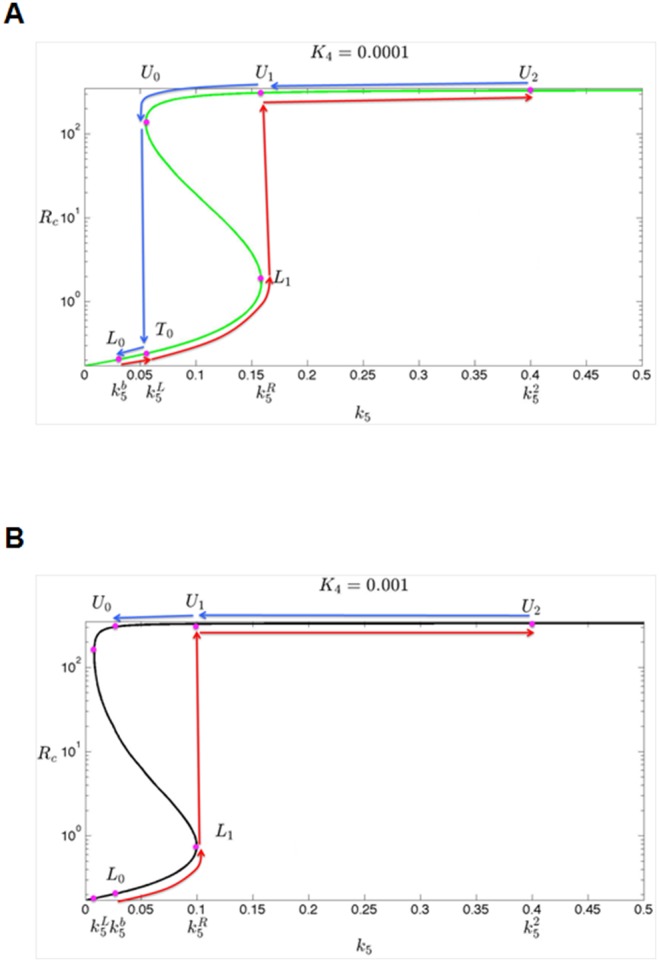
Steady-state bifurcation diagrams. The dependence of the steady states of variable *R*_c_ (RNA level of c-KIT) on parameter *k*_5_ (transcription rate of E2F6) for different values of *K*_4_: (A) *K*_4_ = 0.1 (i.e., low inhibition efficiency of E2F6), (B) *K*_4_ = 1 (i.e., high inhibition efficiency of E2F6). The red and blue arrow line indicates abrupt shifts of the steady state of *R*_c_ with at different *k_5_* values which indicated below. The value of parameters are listed in S2 Text.

In cells with low inhibition efficiency of E2F6 (i.e., a lower *K*_4_), as the parameter *k*_5_ is increased from *k*_5_^b^ to *k*_5_^2^ (red solid line in Fig. 4A), the steady state of *R*_c_ will first move from *L*_0_ to *L*_1_ via the lower branch, and, due to the instability of the steady states in the middle branch, switch from *L*_1_ to *U*_1_ in the upper branch, and finally move along the upper branch to *U*_2_. On the other hand, as the parameter *k*_5_ is decreased from *k*_5_^2^ to *k*_5_^b^ (blue solid line in Fig. 4A), the steady state of *R*_c_ will first move from *U*_2_ to *U*_0_ via the upper branch, and, due to the instability of the steady states in the middle branch, then switch from *U*_0_ to *T*_0_ in the lower branch, and finally move back to *L*_0_. Hence for low inhibition efficiency of E2F6 (i.e., low *K*_4_) and small basal rate constant (i.e., small *k*_5_^b^), addition of estrogen may turn on over-expression of c-KIT mRNA, whereas retraction of estrogen can turn off over-expression of c-KIT mRNA and make the cells recover the normal c-KIT mRNA level.

In cells with high inhibition efficiency of E2F6 (i.e., a higher *K*_4_), as the parameter *k*_5_ is increased from *k*_5_^b^ to *k*_5_^2^ (red solid line in Fig. 4B), the steady state of *R*_c_ will first move from *L*_0_ to *L*_1_ via the lower branch, and, due to the instability of the steady states in the middle branch, switch from *L*_1_ to *U*_1_ in the upper branch, and finally move along the upper branch to *U*_2_. On the other hand, as the parameter *k*_5_ is decreased from *k*_5_^2^ to *k*_5_^b^ (blue solid line in Fig. 4B), the steady state of *R*_c_ will first move from *U*_2_ to *U*_0_ via the upper branch, and, due to the fact that *k*_5_^b^ is the basal transcription rate constant of E2F6 mRNA and *k*_5_^b^ is larger than the *k*_5_ component of left-knee point, then has to stop at *U*_0_ which is the state associated with over-expression of c-KIT mRNA. Hence for high inhibition efficiency of E2F6 (i.e., a higher *K*_4_) and large basal rate constant (i.e., large *k*_5_^b^), retraction of estrogen cannot attenuate the expression of c-KIT mRNA. We make one important observation: for sufficiently large *K*_4_, the *k*_5_ value associated with the left knee is very close to zero. Hence for sufficiently large *K*_4_, it would fit the scenario discussed in this paragraph. This suggests that for sufficiently large *K*_4_, retraction of estrogen cannot switch off over-expression of c-KIT mRNA, which is consistent with the aforementioned clinical observation.

### Validation of the model in TCGA ovarian cancer dataset

**Figure 5:**
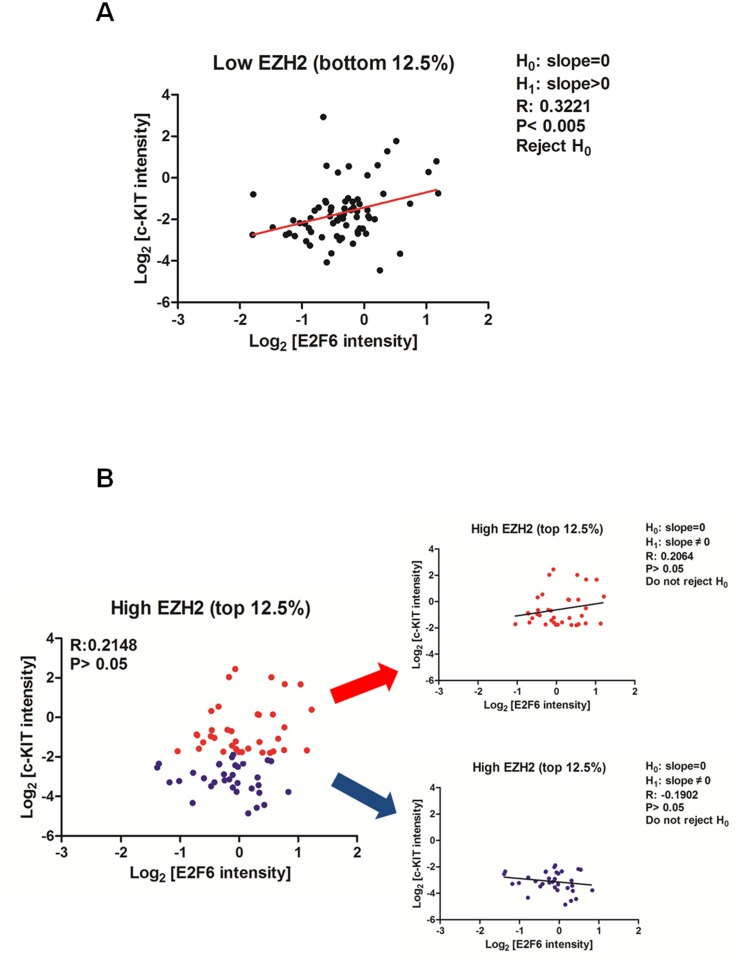
Correlation between expression level of E2F6 and c-KIT in expression microarray dataset from TCGA ovarian cancer patients. Expression microarray data from 568 ovarian cancer patients are sorted according to the expression level of EZH2. The scatter plot demonstrates the correlation between expression level of E2F6 and c-KIT in ovarian cancer patients with (A) low EZH2 (bottom 12.5%) and (B) high EZH2 (top 12.5%). The R and P-value of the Pearson correlation are shown. Interestingly, as predicted by our model, patients with low EZH2 shows a positive correlation of E2F6 and c-KIT (A, R = 0.322, P<0.005). Such correlation is not observed in patients with high EZH2 (B, R = 0.2148. P>0.05). However, patients with high EZH2 can be further separated into 2 sub-groups based on the median value of c-KIT (high, red color; low, blue color). Neither of these sub-groups demonstrates a positive correlation between E2F6 and c-KIT.

In light of the above model, we reasoned that in ovarian cancer patients with low EZH2 (i.e., low *K*_4_), increase of E2F6 mRNA (which results from an increase of transcription rate of E2F6, *k*_5_) may induce the expression of c-KIT mRNA (*R*_c_), through miR-193a-mediated ceRNA mechanism. However, such correlation may not exist in patients with high EZH2 (i.e., high inhibition constant of E2F6 to miR-193a, *K*_4_) as miR-193 is epigenetically silenced. To provide an independent support for this hypothesis, we analyzed the expression microarray data in TCGA ovarian cancer dataset [32]. Interestingly, ovarian cancer patients with low EZH2 level show a positive correlation between the expression of E2F6 and c-KIT (Fig. 5A). Analysis of another set of microarray data in ovarian cancer (GDS2785) also demonstrated a similar phenomenon (S1 Fig.). However, such relationship is not observed in patients with high EZH2 (Fig. 5B and S1 Fig.) or in the correlation between E2F6 and a non-miR-193a target (S2 Fig.). Taken together, this phenomenon is consistent with our prediction (Fig. 3) that mRNA of E2F6 and c-KIT demonstrates a ceRNA relationship at low *K*_4_.

## Discussion

Our mathematical model postulates the existence of a bimodal epigenetic control of miR-193a in ovarian carcinogenesis. In cells with low inhibition efficiency of E2F6, there is a ∼linear relationship between transcription of E2F6 and c-KIT mRNA, thus suggesting a ceRNA relationship between these 2 genes. In this ceRNA hypothesis [28], [29], the 3′UTRs of several mRNAs contain the miR-binding element(s) (MRE) and therefore compete for binding with the miR. Theoretically, a miR can be epigenetically controlled by both promoter hypermethylation and its ceRNA in a bimodal fashion.

**Figure 6:**
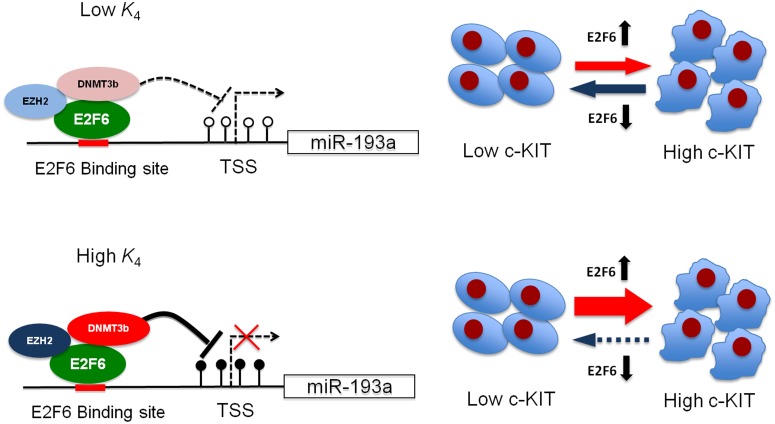
Proposed model for ovarian carcinogenesis. Under the condition of low EZH2 and DNMT3b expression (upper panel, as indicated by light blue and red color), E2F6 only leads to a minimal suppression of miR-193a (i.e., low *K*_4_) and the relationship between E2F6 and c-KIT exhibits a ceRNA relationship. As a result, the amount of c-KIT in the ovarian cancer cells depends on the amount of estrogen (this information is mediated by the level of E2F6). On the contrary, under the condition of high expression level of EZH2 and DNMT3b (lower panel, as indicated by dark blue and red color), E2F6 exerts a high inhibition efficiency (i.e., high *K*_4_). Slight increment of E2F6 results in the epigenetic silencing of miR-193a and subsequently an over-expression of c-KIT. Without miR-193a, the cells may be “locked” in a high c-KIT state resulting in ovarian carcinogenesis.

It is also noteworthy to point out that this ceRNA relationship is a reversible event, such that retraction of estrogen which results in lower expression of E2F6, may reduce the expression of c-KIT (Fig. 6, upper panel). Such phenomenon can be observed in normal ovarian epithelial cells or ovarian cancer cells in which the E2F6 inhibition efficiency is low. Indeed, components of transcriptional repressors such as EZH2 which is required for transcriptional suppression of E2F6 is found to be low in normal ovarian cells and a sub-set of ovarian cancer [33], [34]. Importantly, this is also consistent with the clinical evidence that the expression of E2F6 and c-KIT are correlated in ovarian cancer patients with low expression of EZH2 but not in cases of high EZH2 expression.

However, in cells with high inhibition efficiency of E2F6, our model predicts a highly non-linear relationship between expression of E2F6 and c-KIT. Under the condition of low expression level of E2F6, there follows a ceRNA relationship between E2F6 and c-KIT. However, at high expression of E2F6 together with high expression of transcriptional repressor such as EZH2, E2F6 may lead to epigenetic silencing of the expression of miR-193a through trimethylated H3K27 and DNA methylation (Fig. 6, lower panel). Taken together, these mechanisms may lead to a non-reversible expression of c-KIT and activation of the cell growth signaling in ovarian cancer [35]. This prediction is also consistent with the previous findings that high expression of EZH2 is associated with poor survival in ovarian cancer patients [34]. Inhibition of the EZH2 activity together with anti-estrogen therapy may therefore be effective against ovarian cancer.

In summary, due to the nonlinearities and non-intuitive dynamics of the bimodal regulation of miR-193a, we developed a mathematical model that predicts c-KIT switching behavior. Importantly, a positive correlation of E2F6 and c-KIT is only observed in ovarian cancer patients with low EZH2 expression. Combination treatment of EZH2 inhibitor together with anti-estrogen therapy may be a novel strategy against ovarian cancer.

## Supporting Information

S1 Fig **Correlation between expression level of E2F6 and c-KIT in expression microarray dataset from ovarian cancer patients (GDS2785).** Scatter plot shows the correlation between expression level of E2F6 mRNA and c-KIT in ovarian cancer patients with (A) low EZH2 (n = 16) or (B) high EZH2 (n = 17) according to the median level of EZH2 expression. The R and P-value of the Pearson correlation are also shown. Although not statistically significant, a more positive correlation between expression of c-KIT and EZH2 were observed in patients with low EZH2 (R = 0.3516, P = 0.0766) than that of patients with high EZH2 (R = 0.2938, P = 0.1729).
(TIF)

S2 Fig **Correlation between expression level of E2F6 and CDK5 in expression microarray dataset from TCGA ovarian cancer patients.** To examine if the observation in S1 Fig. is a random event, we analyzed the correlation between expression level of E2F6 and CDK5, a non-miR-193a target in ovarian cancer patients with (A) low EZH2 (bottom 12.5%) and (B) high EZH2 (top 12.5%) as demonstrated in S1 Fig. Positive correlation is not observed in these 2 group of patients (p>0.05).
(TIF)

S1 Table **Description of biological functions of each interaction in** **Fig. 1A** **.**
(PDF)

S1 Text **The existence of steady states.**
(PDF)

S2 Text **The values of parameters in** **Fig. 3** **and** **Fig. 4** **.**
(PDF)

## References

[1] Siegel R, Ma J, Zou Z, Jemal A (2014) Cancer statistics, 2014. CA Cancer J Clin 64:9–29. 24399786 10.3322/caac.21208

[2] Nossov V, Amneus M, Su F, Lang J, Janco JM, et al (2008) The early detection of ovarian cancer: from traditional methods to proteomics. Can we really do better than serum CA-125? Am J Obstet Gynecol 199:215–223. 18468571 10.1016/j.ajog.2008.04.009

[3] Fung-Kee-Fung M, Oliver T, Elit L, Oza A, Hirte HW, et al (2007) Optimal chemotherapy treatment for women with recurrent ovarian cancer. Curr Oncol 14:195–208. 17938703 10.3747/co.2007.148 PMC2002482

[4] Visvader JE, Lindeman GJ (2008) Cancer stem cells in solid tumours: accumulating evidence and unresolved questions. Nat Rev Cancer 8:755–768. 18784658 10.1038/nrc2499

[5] Zhang S, Balch C, Chan MW, Lai HC, Matei D, et al (2008) Identification and characterization of ovarian cancer-initiating cells from primary human tumors. Cancer Res 68:4311–4320. 18519691 10.1158/0008-5472.CAN-08-0364 PMC2553722

[6] Auersperg N, Wong AS, Choi KC, Kang SK, Leung PC (2001) Ovarian surface epithelium: biology, endocrinology, and pathology. Endocr Rev 22:255–288. 11294827 10.1210/edrv.22.2.0422

[7] Cunat S, Hoffmann P, Pujol P (2004) Estrogens and epithelial ovarian cancer. Gynecol Oncol 94:25–32. 15262115 10.1016/j.ygyno.2004.03.026

[8] Weiss NS, Lyon JL, Krishnamurthy S, Dietert SE, Liff JM, et al (1982) Noncontraceptive estrogen use and the occurrence of ovarian cancer. J Natl Cancer Inst 68:95–98. 6948131

[9] Lacey JV Jr, Brinton LA, Leitzmann MF, Mouw T, Hollenbeck A, et al (2006) Menopausal hormone therapy and ovarian cancer risk in the National Institutes of Health-AARP Diet and Health Study Cohort. J Natl Cancer Inst 98:1397–1405. 17018786 10.1093/jnci/djj375

[10] Glud E, Kjaer SK, Thomsen BL, Hogdall C, Christensen L, et al (2004) Hormone therapy and the impact of estrogen intake on the risk of ovarian cancer. Arch Intern Med 164:2253–2259. 15534163 10.1001/archinte.164.20.2253

[11] Beral V, Bull D, Green J, Reeves G (2007) Ovarian cancer and hormone replacement therapy in the Million Women Study. Lancet 369:1703–1710. 17512855 10.1016/S0140-6736(07)60534-0

[12] Baylin SB, Jones PA (2011) A decade of exploring the cancer epigenome - biological and translational implications. Nat Rev Cancer 11:726–734. 21941284 10.1038/nrc3130 PMC3307543

[13] Jones PA, Baylin SB (2002) The fundamental role of epigenetic events in cancer. Nat Rev Genet 3:415–428. 12042769 10.1038/nrg816

[14] Esquela-Kerscher A, Slack FJ (2006) Oncomirs - microRNAs with a role in cancer. Nat Rev Cancer 6:259–269. 16557279 10.1038/nrc1840

[15] Esteller M (2007) Cancer epigenomics: DNA methylomes and histone-modification maps. Nat Rev Genet 8:286–298. 17339880 10.1038/nrg2005

[16] Garzon R, Marcucci G, Croce CM (2010) Targeting microRNAs in cancer: rationale, strategies and challenges. Nat Rev Drug Discov 9:775–789. 20885409 10.1038/nrd3179 PMC3904431

[17] Lujambio A, Calin GA, Villanueva A, Ropero S, Sanchez-Cespedes M, et al (2008) A microRNA DNA methylation signature for human cancer metastasis. Proc Natl Acad Sci U S A 105:13556–13561. 18768788 10.1073/pnas.0803055105 PMC2528872

[18] Gao XN, Lin J, Li YH, Gao L, Wang XR, et al (2011) MicroRNA-193a represses c-kit expression and functions as a methylation-silenced tumor suppressor in acute myeloid leukemia. Oncogene 30:3416–3428. 21399664 10.1038/onc.2011.62

[19] Heller G, Weinzierl M, Noll C, Babinsky V, Ziegler B, et al (2012) Genome-wide miRNA expression profiling identifies miR-9-3 and miR-193a as targets for DNA methylation in non-small cell lung cancers. Clin Cancer Res 18:1619–1629. 22282464 10.1158/1078-0432.CCR-11-2450

[20] Li Y, Gao L, Luo X, Wang L, Gao X, et al (2013) Epigenetic silencing of microRNA-193a contributes to leukemogenesis in t(8;21) acute myeloid leukemia by activating the PTEN/PI3K signal pathway. Blood 121:499–509. 23223432 10.1182/blood-2012-07-444729

[21] Kozaki K, Imoto I, Mogi S, Omura K, Inazawa J (2008) Exploration of tumor-suppressive microRNAs silenced by DNA hypermethylation in oral cancer. Cancer Res 68:2094–2105. 18381414 10.1158/0008-5472.CAN-07-5194

[22] Carroll JS, Meyer CA, Song J, Li W, Geistlinger TR, et al (2006) Genome-wide analysis of estrogen receptor binding sites. Nat Genet 38:1289–1297. 17013392 10.1038/ng1901

[23] Chen HZ, Tsai SY, Leone G (2009) Emerging roles of E2Fs in cancer: an exit from cell cycle control. Nat Rev Cancer 9:785–797. 19851314 10.1038/nrc2696 PMC3616489

[24] Trimarchi JM, Fairchild B, Wen J, Lees JA (2001) The E2F6 transcription factor is a component of the mammalian Bmi1-containing polycomb complex. Proc Natl Acad Sci U S A 98:1519–1524. 11171983 10.1073/pnas.041597698 PMC29289

[25] Attwooll C, Oddi S, Cartwright P, Prosperini E, Agger K, et al (2005) A novel repressive E2F6 complex containing the polycomb group protein, EPC1, that interacts with EZH2 in a proliferation-specific manner. J Biol Chem 280:1199–1208. 15536069 10.1074/jbc.M412509200

[26] Velasco G, Hube F, Rollin J, Neuillet D, Philippe C, et al (2010) Dnmt3b recruitment through E2F6 transcriptional repressor mediates germ-line gene silencing in murine somatic tissues. Proc Natl Acad Sci U S A 107:9281–9286. 20439742 10.1073/pnas.1000473107 PMC2889045

[27] Gerstein MB, Kundaje A, Hariharan M, Landt SG, Yan KK, et al (2012) Architecture of the human regulatory network derived from ENCODE data. Nature 489:91–100. 22955619 10.1038/nature11245 PMC4154057

[28] Salmena L, Poliseno L, Tay Y, Kats L, Pandolfi PP (2011) A ceRNA hypothesis: the Rosetta Stone of a hidden RNA language? Cell 146:353–358. 21802130 10.1016/j.cell.2011.07.014 PMC3235919

[29] Poliseno L, Salmena L, Zhang J, Carver B, Haveman WJ, et al (2010) A coding-independent function of gene and pseudogene mRNAs regulates tumour biology. Nature 465:1033–1038. 20577206 10.1038/nature09144 PMC3206313

[30] Makar AP (2000) Hormone therapy in epithelial ovarian cancer. Endocr Relat Cancer 7:85–93. 10903526 10.1677/erc.0.0070085

[31] Smyth JF, Gourley C, Walker G, MacKean MJ, Stevenson A, et al (2007) Antiestrogen therapy is active in selected ovarian cancer cases: the use of letrozole in estrogen receptor-positive patients. Clin Cancer Res 13:3617–3622. 17575226 10.1158/1078-0432.CCR-06-2878

[32] Network TR (2011) Integrated genomic analyses of ovarian carcinoma. Nature 474:609–615. 21720365 10.1038/nature10166 PMC3163504

[33] Li H, Cai Q, Godwin AK, Zhang R (2010) Enhancer of zeste homolog 2 promotes the proliferation and invasion of epithelial ovarian cancer cells. Mol Cancer Res 8:1610–1618. 21115743 10.1158/1541-7786.MCR-10-0398 PMC3059727

[34] Lu C, Han HD, Mangala LS, Ali-Fehmi R, Newton CS, et al (2010) Regulation of tumor angiogenesis by EZH2. Cancer Cell 18:185–197. 20708159 10.1016/j.ccr.2010.06.016 PMC2923653

[35] Chau WK, Ip CK, Mak AS, Lai HC, Wong AS (2013) c-Kit mediates chemoresistance and tumor-initiating capacity of ovarian cancer cells through activation of Wnt/beta-catenin-ATP-binding cassette G2 signaling. Oncogene 32:2767–2781. 22797058 10.1038/onc.2012.290

