# Supplementary figures and images for: A Mathematical Model of Bimodal Epigenetic Control of miR-193a in Ovarian Cancer Stem Cells
Source: PLoS One. 2014 Dec 29;9(12):e116050. doi: 10.1371/journal.pone.0116050 (PMC4278842; doi:10.1371/journal.pone.0116050)

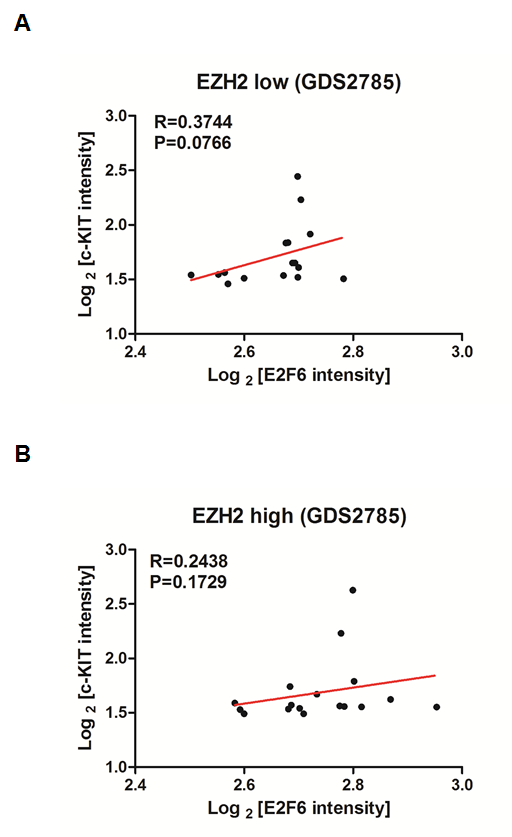

Supplement: S1 Fig — Correlation between expression level of E2F6 and c-KIT in expression microarray dataset from ovarian cancer patients (GDS2785). Scatter plot shows the correlation between expression level of E2F6 mRNA and c-KIT in ovarian cancer patients with (A) low EZH2 (n = 16) or (B) high EZH2 (n = 17) according to the median level of EZH2 expression. The R and P-value of the Pearson correlation are also shown. Although not statistically significant, a more positive correlation between expression of c-KIT and EZH2 were observed in patients with low EZH2 (R = 0.3516, P = 0.0766) than that of patients with high EZH2 (R = 0.2938, P = 0.1729). (TIF) [file pone.0116050.s001.tif]

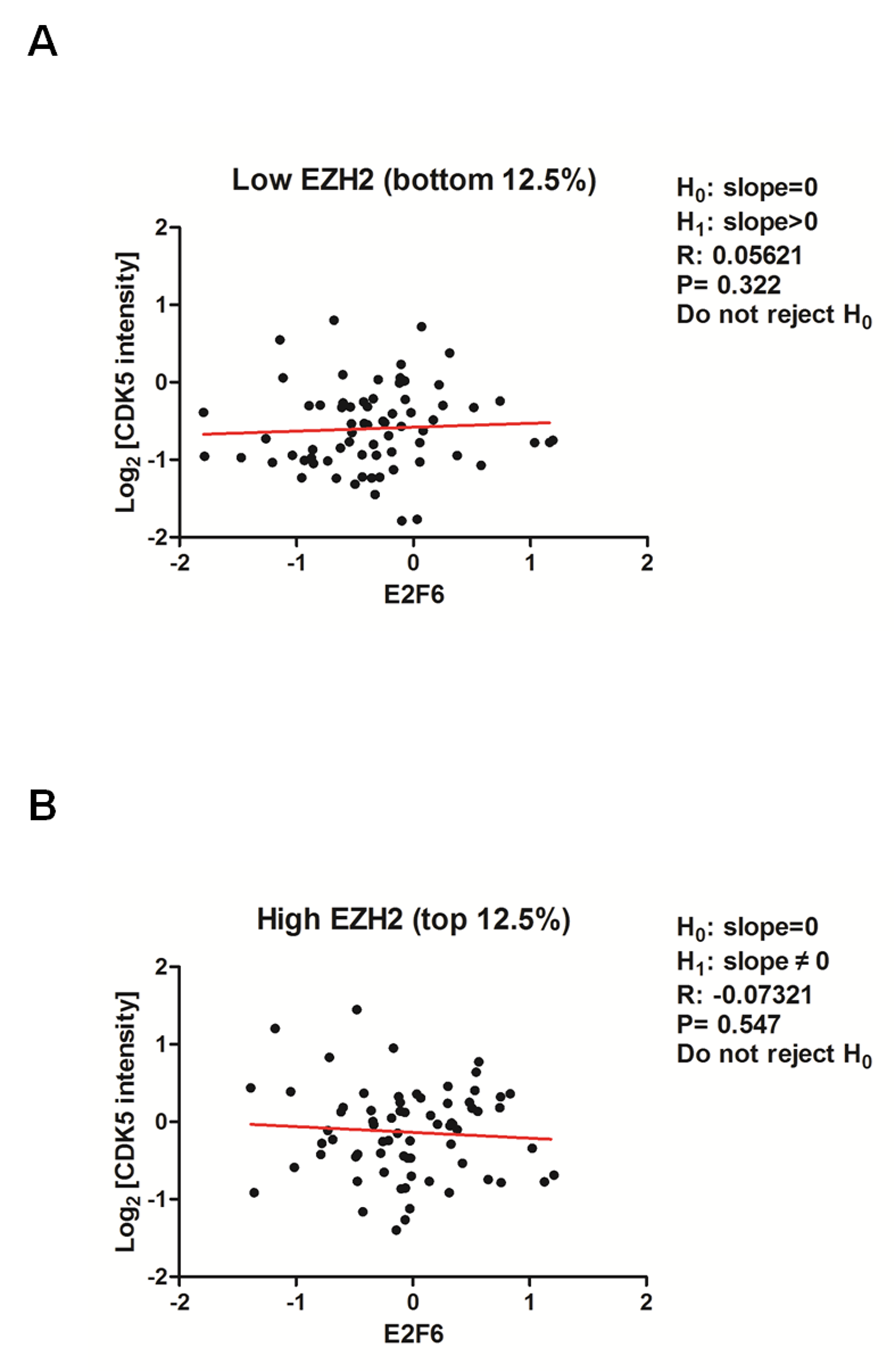

Supplement: S2 Fig — Correlation between expression level of E2F6 and CDK5 in expression microarray dataset from TCGA ovarian cancer patients. To examine if the observation in S1 Fig. is a random event, we analyzed the correlation between expression level of E2F6 and CDK5, a non-miR-193a target in ovarian cancer patients with (A) low EZH2 (bottom 12.5%) and (B) high EZH2 (top 12.5%) as demonstrated in S1 Fig. Positive correlation is not observed in these 2 group of patients (p>0.05). (TIF) [file pone.0116050.s002.tif]
