# Supplementary material for: A Mathematical Model of Bimodal Epigenetic Control of miR-193a in Ovarian Cancer Stem Cells
Source: PLoS One. 2014 Dec 29;9(12):e116050. doi: 10.1371/journal.pone.0116050 (PMC4278842; doi:10.1371/journal.pone.0116050)
Supplement: S1 Table — Description of biological functions of each interaction in Fig. 1A . (PDF) [file pone.0116050.s003.pdf]

Table S1: Description of the biological function of each pathway in Fig 1A

**Interaction Number in Fig 1a**	**Description of Function**	**References**
1 (E2F6 DNA→RNA)	"E2F6 DNA transcribes to E2F6 mRNA."	
2 (c-KIT DNA→RNA)	"c-KIT DNA transcribes to c-KIT mRNA."	
3 (miR193a DNA→RNA)	"miR193a DNA transcribes to miR193a RNA."	
4 (miR193a--|E2F6)	"miR193a inhibits translation of E2F6."	(1)
5 (miR193a--|c-KIT)	"miR193a inhibits translation of c-KIT."	(2-3)
6 (E2F6--|miR193a)	"The knockdown mechanism of endogenous miR193a is carried out by E2F6 over-expression, which acted like a sponge in absorbing the miR193a."	Proposed
7 (c-KIT --|miR193a)	"The knockdown mechanism of endogenous miR193a is carried out by c-KIT over-expression, which acted like a sponge absorbing the miR193a."	Proposed
8 (E2F6 RNA→protein)	"E2F6 RNA translates to E2F6 RNA."	
9 (E2F6--|miR193a)	"E2F6 may lead to epigenetic silencing of miR-193a."	(4-6)

References

1. Kozaki K, Imoto I, Mogi S, Omura K, & Inazawa J (2008) Exploration of tumor-suppressive microRNAs silenced by DNA hypermethylation in oral cancer. *Cancer Res* 68(7):2094-2105.
2. Gao XN, *et al.* (2011) MicroRNA-193a represses c-kit expression and functions as a methylation-silenced tumor suppressor in acute myeloid leukemia. *Oncogene* 30(31):3416-3428.
3. Li Y, *et al.* (2013) Epigenetic silencing of microRNA-193a contributes to leukemogenesis in t(8;21) acute myeloid leukemia by activating the PTEN/PI3K signal pathway. *Blood* 121(3):499-509.
4. Trimarchi JM, Fairchild B, Wen J, & Lees JA (2001) The E2F6 transcription factor is a component of the mammalian Bmi1-containing polycomb complex. *Proc Natl Acad Sci U S A* 98(4):1519-1524.
5. Attwooll C, *et al.* (2005) A novel repressive E2F6 complex containing the polycomb group protein, EPC1, that interacts with EZH2 in a proliferation-specific manner. *J Biol Chem* 280(2):1199-1208.
6. Velasco G, *et al.* (2010) Dnmt3b recruitment through E2F6 transcriptional repressor mediates germ-line gene silencing in murine somatic tissues. *Proc Natl Acad Sci U S A* 107(20):9281-9286.
7. Carla Bosia, Andrea Pagnani, Riccardo Zecchina (201x) Modelling competing endogenous RNA networks - Supporting Information
