# Supplementary material for: A Mathematical Model of Bimodal Epigenetic Control of miR-193a in Ovarian Cancer Stem Cells
Source: PLoS One. 2014 Dec 29;9(12):e116050. doi: 10.1371/journal.pone.0116050 (PMC4278842; doi:10.1371/journal.pone.0116050)
Supplement: S1 Text — The existence of steady states. (PDF) [file pone.0116050.s004.pdf]

Supplementary Text S1

The existence of steady states

The steady states of system (1)-(6) are given by equating the right-hand sides of system (1)-(6) to zero, and thus governed by the following equations:

$$\frac{k_4}{1+K_4P} + k_{m1}R_{em} + k_{m2}R_{mc} = k_1R_mR_e + k_2R_mR_c + \delta_mR_m \tag{S1}$$

$$k_5 + k_{m1}R_{em} = k_1R_mR_e + \delta_eR_e \tag{S2}$$

$$k_6 + k_{m2}R_{mc} = k_2R_mR_c + \delta_cR_c \tag{S3}$$

$$k_1R_mR_e = k_{m1}R_{em} + \delta_{em}R_{em} \tag{S4}$$

$$k_2R_mR_c = k_{m2}R_{mc} + \delta_{mc}R_{mc} \tag{S5}$$

$$k_3R_e = \delta_pP \tag{S6}$$

With a direct computation, the above algebraic system gives the expressionof the steady state in terms of R_m as follows:

$$R_e = \frac{k_5A_e}{\delta_eA_e + R_m}$$

$$R_c = \frac{k_6A_c}{\delta_cA_c + R_m}$$

$$R_{em} = \frac{k_1R_mR_e}{k_{m1} + \delta_e}$$

$$R_{mc} = \frac{k_2R_mR_c}{k_{m2} + \delta_c}$$

$$P = \frac{Mk_5A_e}{R_m + D_e}$$

where

$$A_e = \frac{k_{m1}+\delta_{em}}{k_1\delta_{em}}, A_c = \frac{k_{m2}+\delta_{mc}}{k_2\delta_{mc}}, D_e = \delta_eA_e, \text{ and } M = \frac{k_3}{\delta_P}$$

Substituting these expressions into (S1) and rearranging the resulting equation, we obtain

$$k_4 = h(R_m) \tag{S7}$$

where

$$h(x) = \left(\delta_m x + \frac{k_5 x}{x+\delta_e A_e} + \frac{k_6 x}{x+\delta_c A_c}\right)\frac{x+D_e+MA_eK_4k_5}{x+D_e} \quad \text{(S8)}$$

Since $h(0) = 0$ and $h(\infty) = \infty$; for any given $k_4 > 0$; there always exists $R_m^* > 0$ such that $k_4 = h(R_m^*)$.This shows that for system (1)-(6), positive steady states always exist.

The existence of multiple steady states

Next we give a condition for the existence of multiple steady states. Indeed,differentiating (S8) yields

$$h^{'}(x) = \frac{x+D_e+C}{x+D_e}\left(\delta_m + \frac{k_5 D_e}{(x+D_e)^2} + \frac{k_6 D_c}{(x+D_c)^2} - \frac{Ch(x)}{(x+D_e+C)^2}\right) \quad \text{(S9)}$$

whereC $= MA_eK_4k_5$, $D_e = \delta_e A_e$ and $D_c = \delta_c A_c$. Hence, $h^{'}(x) \geq 0$ if and only if

$$\delta_m + \frac{k_5 D_e}{(x+D_e)^2} + \frac{k_6 D_c}{(x+D_c)^2} \geq \frac{Ch(x)}{(x+D_e+C)^2}$$

or, equivalently, by

$$\frac{1}{C} \geq \frac{1}{x+D_e}\frac{\frac{k_5(x^2-D_e^2)}{(x+D_e)^2}+\frac{k_6(x^2-D_eD_c)}{(x+D_c)^2}-\delta_m D_e}{(x+D_e)\left(\delta_m+\frac{k_5 D_e}{(x+D_e)^2}+\frac{k_6 D_c}{(x+D_c)^2}\right)} \equiv l(x) \quad \text{(S10)}$$

Hence, if $k_5 + k_6 > \delta_m D_e$ and $\frac{1}{C} < \max_{x\geq 0} l(x)$,then $h^{'}$ has two consecutive positive zeros, say $0 < x_1 < x_2$. In this case, if k_4 lies between $h(x_1)$ and $h(x_2)$, then system (1)-(6) admits three positive steady states.

Stability Analysis of system (1)-(6)

Finally, by evaluating the determinant of corresponding Jacobian matrix, we establish a sufficient condition on the instability for a given positive steadystate of system(1)-(6). The Jacobian matrix $J$of system (1)-(6) evaluated at $(R_m^*, R_e^*, R_c^*, R_{em}^*, R_{mc}^*, P^*)$ takes the form

$$\begin{bmatrix} -(k_1R_e^* + k_2R_c^* + \delta_m) & -k_1R_m^* & -k_2R_m^* & k_{m1} & k_{m2} & -\frac{k_4K_4}{(1+K_4P^*)^2} \\ -k_1R_e^* & -(k_1R_m^* + \delta_e) & 0 & k_{m1} & 0 & 0 \\ -k_2R_c^* & 0 & -(k_2R_m^* + \delta_c) & 0 & k_{m2} & 0 \\ k_1R_e^* & k_1R_m^* & 0 & -(k_{m1}+\delta_{em}) & 0 & 0 \\ k_2R_c^* & 0 & k_2R_m^* & 0 & -(k_{m2}+\delta_{mc}) & 0 \\ 0 & k_3 & 0 & 0 & 0 & -\delta_P \end{bmatrix}$$

With a direct computation, the determinant $\det(J)$ of J takes

$$det(J) = \begin{vmatrix} \delta_m & 0 & 0 & \delta_{em} & \delta_{mc} & \frac{k_4K_4}{(1+K_4P^*)^2} \\ 0 & \delta_e & 0 & \delta_{em} & 0 & 0 \\ 0 & 0 & \delta_c & 0 & \delta_{mc} & 0 \\ -k_1R_e^* & -k_1R_m^* & 0 & k_{m1}+\delta_{em} & 0 & 0 \\ -k_2R_c^* & 0 & -k_2R_m^* & 0 & k_{m2}+\delta_{mc} & 0 \\ 0 & -k_3 & 0 & 0 & 0 & \delta_p \end{vmatrix}$$

From Eqs.(S2)- (S5),we have $k_5 = \delta_e R_e^* + \delta_{em} R_{em}^*$ and $k_6 = \delta_c R_c^* + \delta_{mc} R_{mc}^*$ .Together with relations (S4)-(S5), $\det(J)$is reduced to the form

d

$$det(J) = \frac{k_1k_2R_m^*}{R_{em}^* R_{mc}^*} \begin{vmatrix} \delta_m R_m^* & 0 & 0 & \delta_{em} R_{em}^* & \delta_{mc} R_{mc}^* & \frac{k_4K_4}{(1+K_4P^*)^2} \\ 0 & \delta_e R_e^* & 0 & \delta_{em} R_{em}^* & 0 & 0 \\ 0 & 0 & \delta_c R_c^* & 0 & \delta_{mc} R_{mc}^* & 0 \\ 1 & 1 & 0 & -1 & 0 & 0 \\ 1 & 0 & 1 & 0 & -1 & 0 \\ 0 & -k_3R_e^* & 0 & 0 & 0 & \delta_p \end{vmatrix}$$

$$= \frac{k_1k_2R_m^*}{R_{em}^* R_{mc}^*} \begin{vmatrix} \delta_m R_m^* & -\delta_e R_e^* + \frac{k_4K_4}{(1+K_4P^*)^2}\frac{k_3R_e^*}{\delta_P} & -\delta_c R_c^* & 0 & 0 & \frac{k_4K_4}{(1+K_4P^*)^2} \\ \delta_{em} R_{em}^* & k_5 & 0 & \delta_{em} R_{em}^* & 0 & 0 \\ \delta_{mc} R_{mc}^* & 0 & k_6 & 0 & \delta_{mc} R_{mc}^* & 0 \\ 0 & 0 & 0 & -1 & 0 & 0 \\ 0 & 0 & 0 & 0 & -1 & 0 \\ 0 & 0 & 0 & 0 & 0 & \delta_p \end{vmatrix}$$

$$= \frac{\delta_P k_1k_2k_5k_6R_m^*}{R_{em}^* R_{mc}^*}\left(\delta_m R_m^* + \frac{\delta_c R_c^* \delta_{mc} R_{mc}^*}{k_6} + \frac{\delta_e R_e^* \delta_{em} R_{em}^*}{k_5} - \frac{\delta_{em} R_{em}^*}{k_5}\frac{k_4K_4}{(1+K_4P^*)^2}\frac{k_3R_e^*}{\delta_P}\right)$$

Recall that R_e^* , R_c^* , R_{em}^* , R_{mc}^* , $P^*$are expressed in terms of R_m^*. Hence $\det(J)$can be reduced to the following form:

$$\det(J)=\frac{\delta_P k_1 k_2 k_5 k_6 (R_m^*)^2}{R_{em}^* R_{mc}^*}\left(\delta_m+\frac{k_6\delta_c A_c}{(R_m^*+\delta_c A_c)^2}+\frac{k_5\delta_e A_e}{(R_m^*+\delta_e A_e)^2}-\frac{\frac{k_3 K_4 k_5 A_e}{\delta_p}\cdot k_4}{(R_m^*+D_e+C)^2}\right)$$

Finally, using the relation $k_4=h(R_m^*)$ and (S9), we arrive at the identity

$$\det(J)=\frac{\delta_P k_1 k_2 k_5 k_6 (R_m^*)^2}{R_{em}^* R_{mc}^*}\frac{R_m^*+D_e}{R_m^*+D_e+C}h'(R_m^*).$$

Hence, if $h'(R_m^*)<0$ then the corresponding positive steady state is unstable. An immediate application of this result is that the steady states of the middle branch are always unstable.
