# Supplementary material for: A Mathematical Model of Bimodal Epigenetic Control of miR-193a in Ovarian Cancer Stem Cells
Source: PLoS One. 2014 Dec 29;9(12):e116050. doi: 10.1371/journal.pone.0116050 (PMC4278842; doi:10.1371/journal.pone.0116050)
Supplement: S2 Text — The values of parameters in Fig. 3 and Fig. 4 . (PDF) [file pone.0116050.s005.pdf]

Supplementary Text S2

The values of kinetic parameters used in Figures 3 and 4 are given in the following list.

Synthesis-related rates:

miR193a transcription rate: $k_4 = 0.3\text{molecule} \cdot \text{sec}^{-1}$;

c-KIT mRNA transcription rate: $k_6 = 0.1\text{molecule} \cdot \text{sec}^{-1}$;

E2F6 protein translation rate: $k_3 = 0.03\text{molecule} \cdot \text{sec}^{-1}$;

- E2F6 mRNA transcription rate: k_5;
- Inhibition strength of miR193a expression: K_4.

} **control/ bifurcation parameters**

Degradation rates:

miR193a degradation rate: $\delta_\text{m} = 0.0001\ \text{sec}^{-1}$;

E2F6 mRNA degradation rate: $\delta_\text{e} = 0.0003\text{sec}^{-1}$;

c-KIT mRNA degradation rate: $\delta_\text{c} = 0.0003\text{sec}^{-1}$;

miR193a–E2F6 mRNA complex degradation rate: $\delta_\text{em} = 0.003\text{sec}^{-1}$;

miR193a–c-KIT mRNA complex degradation rate: $\delta_\text{mc} = 0.003\text{sec}^{-1}$;

E2F6 protein degradation rate: $\delta_\text{p} = 0.0001\text{sec}^{-1}$.

Complexes – binding and unbinding rates:

miR193a & E2F6 mRNA association rate: $k_1 = 0.0003\text{sec}^{-1}\text{molecule}^{-1}$;

miR193a & c-KIT mRNA association rate: $k_2 = 0.0003\text{sec}^{-1}\text{molecule}^{-1}$;

miR193a–E2F6 mRNA complex dissociation rate: $k_{\text{m}1} = 0.0001\text{sec}^{-1}$;

miR193a–c-KIT mRNA complex dissociation rate: $k_{\text{m}2} = 0.0001\text{sec}^{-1}$.
